# Radiolocalization of atypical lesions for intraoperative identification: technical factors, localization quality, success rates, patient safety, and spectrum of applications

**DOI:** 10.1186/s12957-019-1631-7

**Published:** 2019-05-27

**Authors:** Jason R. Young, Andi E. Wallig, Nichole L. Fischer, Tiffinee N. Swanson, Mark J. Truty, K. Robert Shen, Brendan P. McMenomy

**Affiliations:** 1 Department of Radiology, Mayo Clinic, 200 First Street SW, Rochester, MN 55905 USA; 2 Department of Hepatobiliary/Pancreatic Surgery, Mayo Clinic, 200 First Street SW, Rochester, MN 55905 USA; 3 Department of Thoracic Surgery, Mayo Clinic, 200 First Street SW, Rochester, MN 55905 USA

**Keywords:** Radiolocalization, Injection, Intraoperative, Localization

## Abstract

**Background:**

To retrospectively analyze perilesional technetium Tc-99m MAA injection for intraoperative localization of atypical soft-tissue and bone lesions within a single tertiary referral center in order to determine technique, safety, and clinical utility of these procedures.

**Methods:**

An IRB compliant, retrospective electronic chart review (2010–2017) exploring surgical excision of atypical (non-pulmonary, non-breast, non-sentinel node) lesions guided by Tc-99m MAA perilesional injection. Patient demographics, lesion location, lesion size, radiotracer injection technique, radiotracer injection complications, scintigraphy technique, scintigraphic quality, intraoperative time, lesion identification in surgery, and pathological diagnoses were recorded.

**Results:**

Twenty-two atypical radiolocalization exams were identified. Lesion sites included rib (7), lymph node (4), abdominal wall (3), mesenteric (3), gallbladder fossa (1), retroperitoneum (1), parietal pleura (1), anterior mediastinum (1), and iliac bone (1). Average lesion size was 14 mm (range 5–23 mm). Eighteen (82%) radiotracer injections used computed tomography guidance and 4 (18%) used ultrasound guidance. The mean activity of Tc-99m MAA administered was 11.8 MBq (0.32 mCi). A 22-gauge needle was most often used for perilesional injection. No injection complications were reported. The lesions were identified with a hand-held gamma probe during surgery in 100% of cases. Of the samples sent to pathology, 100% were identified and given a diagnosis.

**Conclusion:**

Radiolocalization of atypical lesions may be a valuable technique, guiding minimally invasive surgical removal of lesions that would otherwise be difficult to identify intraoperatively such as non-palpable rib, central mesenteric nodal, and abdominal wall lesions.

## Background

Radiolocalization is a technique that most commonly utilizes a small volume (0.2–1.0 cc) of technetium 99m labeled macroaggregated albumin (Tc-99m MAA) or sulfur colloid (Tc-99m SC) to localize lymph nodes and lesions throughout the body. Intraoperatively, a small hand-held gamma probe can then be used for confirmation of the lesion prior to excision. There are two major facets of radiolocalization: intradermal injection for sentinel lymph node localization and direct perilesional injections.

This study evaluates technique, safety, and clinical utility of perilesional radiotracer injections for intraoperative localization: a technique that utilizes ultrasound or computed tomography (CT) to visualize a lesion, guide a needle to it, and inject Tc-99m MAA. Perilesional Tc-99m MAA remains near the lesion for several hours, allowing subsequent intraoperative localization. The application of perilesional injections for small and non-palpable pulmonary lesions is well described [1–4]. There are several reports on perilesional breast radiolocalization, with the benefit of simultaneously localizing non-palpable breast lesions and sentinel lymph nodes [5–8]. While other applications of perilesional localizations have been described, the information is limited.

## Methods

The aims of this study were to retrospectively review perilesional radiolocalization data from Mayo Clinic Rochester (a tertiary referral center) in order to (1) describe the spectrum of applications, (2) define injection and imaging techniques, (3) evaluate risk to patients, (4) review the quality of associated scintigraphic images, and (5) determine clinical success.

An IRB-approved retrospective review of the Mayo Clinic Rochester institutional database was performed. A list of 897 potential subjects was created using ICD 9 and 10 codes for “Nuclear Medicine Lesion Localization” exams performed between November 1, 2002, and November 1, 2017. Exam descriptors and reports were then reviewed to exclude pulmonary, cutaneous, and breast radiotracer localizations.

Data collected from electronic medical record review of the final subjects included sex, age at time of radiolocalization, anatomic location and size of lesion, modality (CT versus ultrasound) utilized to guide radiotracer injection, needle gauge for injection, radioactivity of Tc-99m MAA administered, patient complications associated with radiotracer injection, post-injection scintigraphy parameters (matrix, acquisition time, total counts, collimator type), surgery time for lesion excision, successful intraoperative lesion localization with a hand-held gamma probe, lesion identification, and diagnosis from the pathology service.

Scintigraphic images were reviewed by two board-certified radiologists. A grading system was developed to quantify the quality of radiotracer localization based on post-injection scintigraphy (1 = focal on target without surrounding activity, 2 = focal on target with mild surrounding activity, 3 = focal on target with moderate surrounding activity, 4 = focal or non-focal in general region of target, 5 = absent or outside target region). The presence of radiotracer draining into adjacent lymph nodes was also recorded.

JMP software for Macintosh (SAS Institute Inc. Cary, NC, USA) was utilized for data analysis. Distribution of continuous variables was expressed as a mean and range. A *P* value <0.05 was established as statistically significant. The Fisher exact test was used for between-group comparison of categorical variables and an unpaired Student *t* test was utilized for between-group comparison of continuous variables.

## Results

Twenty-two patients were identified: 9 females and 13 males. Anatomic sites of lesions included rib (7), superficial lymph node (4), mesenteric lymph node (3), abdominal wall (3), gallbladder fossa (1), retroperitoneum (1), parietal pleura (1), anterior mediastinum (1), and iliac bone (1). The average lesion size was 14 mm (range 5–23 mm).

All radiotracer injections were performed on the day of surgery by physicians skilled in radiologic interventional procedures. Eighteen (82%) radiotracer injections used CT guidance and 4 (18%) used ultrasound guidance. The mean activity of unfiltered Tc-99m MAA administered was 11.8 MBq (0.32 mCi) with a range of 11.1–18.5 MBq (0.30–0.50 mCi). The volume of radiotracer was 0.3 mL, injected with a 1 mL tuberculin syringe, attached to a stylet needle system. The needle gauge used most often was 22 (45%, 10/22) followed by 19 (23%, 5/22) with a range of 17–25. No injection complications such as hemorrhage or bowel perforation were reported.

All post-injection scintigrams had both anterior-posterior and lateral views. All images were acquired on a 128 × 128 matrix immediately after radiotracer injection. Scintigraphy collimators utilized 18 (82%) low energy high resolution, 3 (13.5%) medium energy general purpose, and 1 (4.5%) low energy general purpose. The mean number of counts acquired during anterior imaging was 81,232 (range 22,068–210,508) with an average acquisition time of 1.43 min (range 0.57–3.44 min). The mean number of counts acquired during lateral imaging was 120,300 (range 50,928–222,593) with an average acquisition time of 1.34 min (range 0.34–4.11 min).

A high quality of radiotracer localization by scintigraphy was present in a majority of cases with 86% (19/22) scoring a 1, while 9% (2/22) of cases scored a 2, and 5% (1/22) scored a 3. There were no cases of poor scintigraphy localization scores of 4 or 5. The cases with a score of 2 were retroperitoneal and anterior rib lesions. The case with a score of 3 was an anterior rib lesion. Three cases had tracer activity in adjacent solitary draining lymph nodes.

The average length of surgery was 140 min (range 23–517 min). Surgical excision of the localized lesion was combined with other synchronous surgical procedures at times. The lesion was identified with a standard hand-held gamma probe during surgery in 100% of cases, often allowing for a smaller incision or more minimally invasive approach. All but two cases had samples sent to pathology. Of the 20 samples sent to pathology, 45% (9/20) received a diagnosis of metastatic disease and the remaining 55% (11/20) were given a diagnosis of infection or inflammation.

Based on chart review by a surgeon, 91% (20/22) of the cases clearly had favorable clinical impact by utilizing perilesional radiolocalization. There were no significant correlations or even trends toward the correlation between the various data collected such as localization quality and imaging or injection technique.

## Discussion

**Fig. 1:**
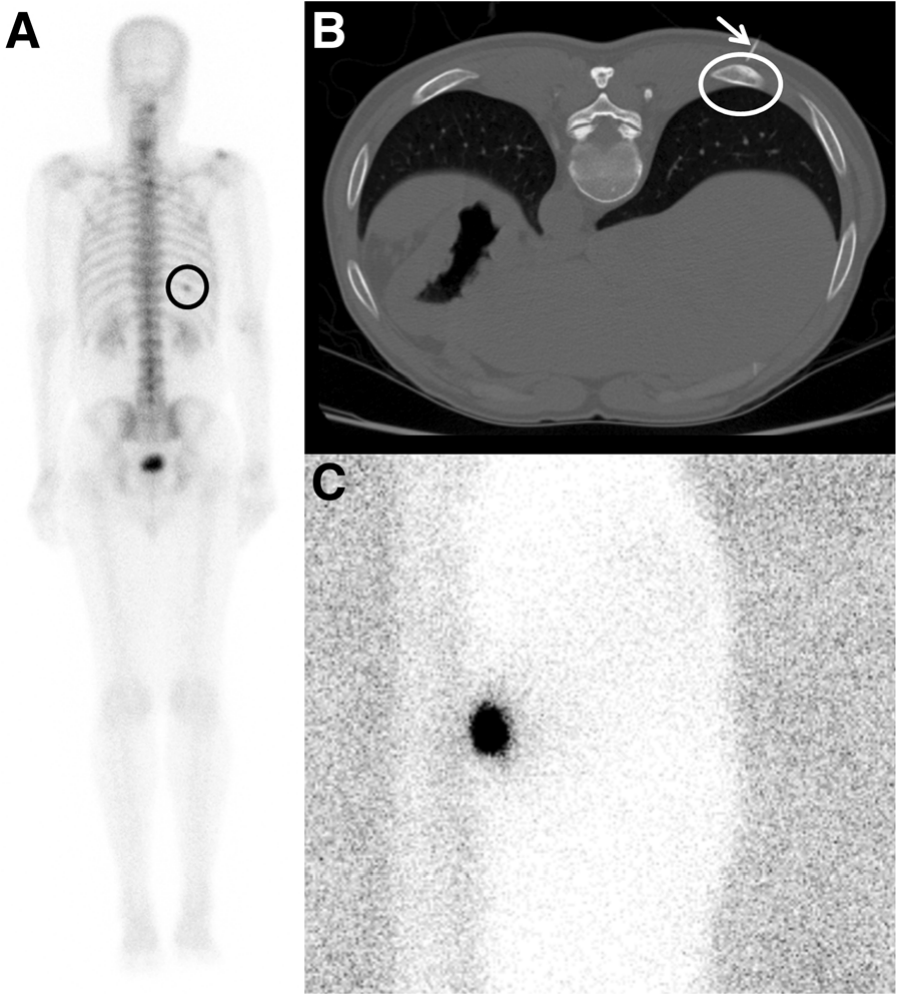
A 60-year-old male, with history of treated Gleason 7 prostate carcinoma presenting with a rising prostate-specific antigen (PSA). The only active site of disease was a posterior right 10th rib lesion (black circle) identified on a Tc-99m methyl diphosphonate bone scan (**a**). This lesion underwent CT-guided radiolocalization (**b**) where a 9-mm sclerotic rib lesion (white circle) is being injected with Tc-99m MAA using a percutaneous needle (white arrow). A right lateral scintigram (**c**) depicts focal radiotracer in the region of targeted rib lesion. This portion of the right 10th rib was surgically excised and pathology confirmed metastatic prostatic adenocarcinoma. Patient’s serum PSA subsequently dropped from 29.3 to 3.7 ng/mL

**Fig. 2:**
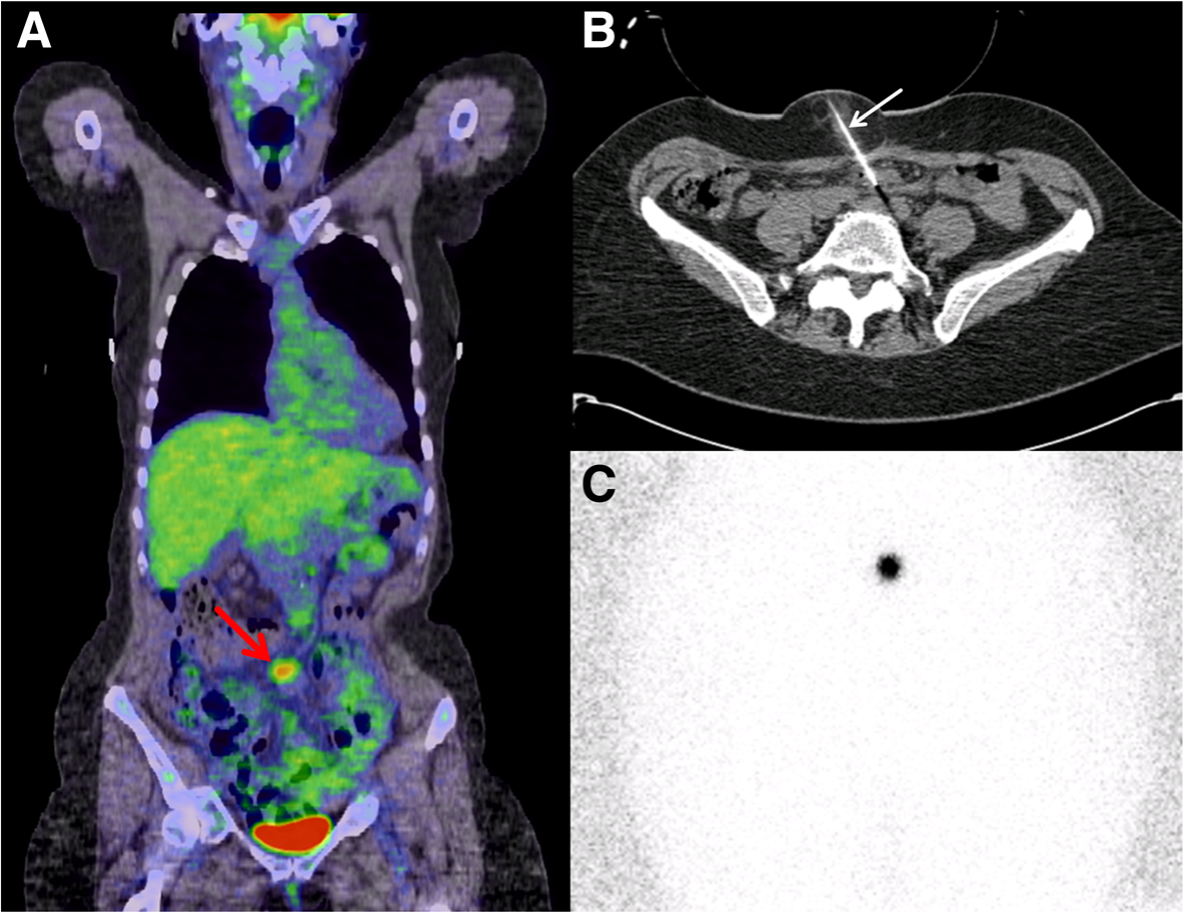
A 72-year-old female with high-grade serous ovarian carcinoma. Six years after surgical resection and chemotherapy, rising serum CA-125 prompted a F-18 fluorodeoxyglucose positron emission tomography/computed tomography (FDG PET/CT) exam. Coronal fusion FDG PET/CT (**a**) depicts a hypermetabolic lymph node in her mesentery (red arrow) that was suspicious as a solitary site for metastatic disease. Given concern for difficulty palpating this node intraoperatively, radiolocalization was performed. Using CT guidance (**b**), a compression device with hollow center displaces overlying bowel as the Tc-99m MAA injection needle (white arrow) is advanced to the target lymph node for perilesional injection. Excellent localization was confirmed by anterior pelvic scintigram (**c**). This lymph node was surgically removed and confirmed metastatic disease, prompting initiation of chemotherapy

**Fig. 3:**
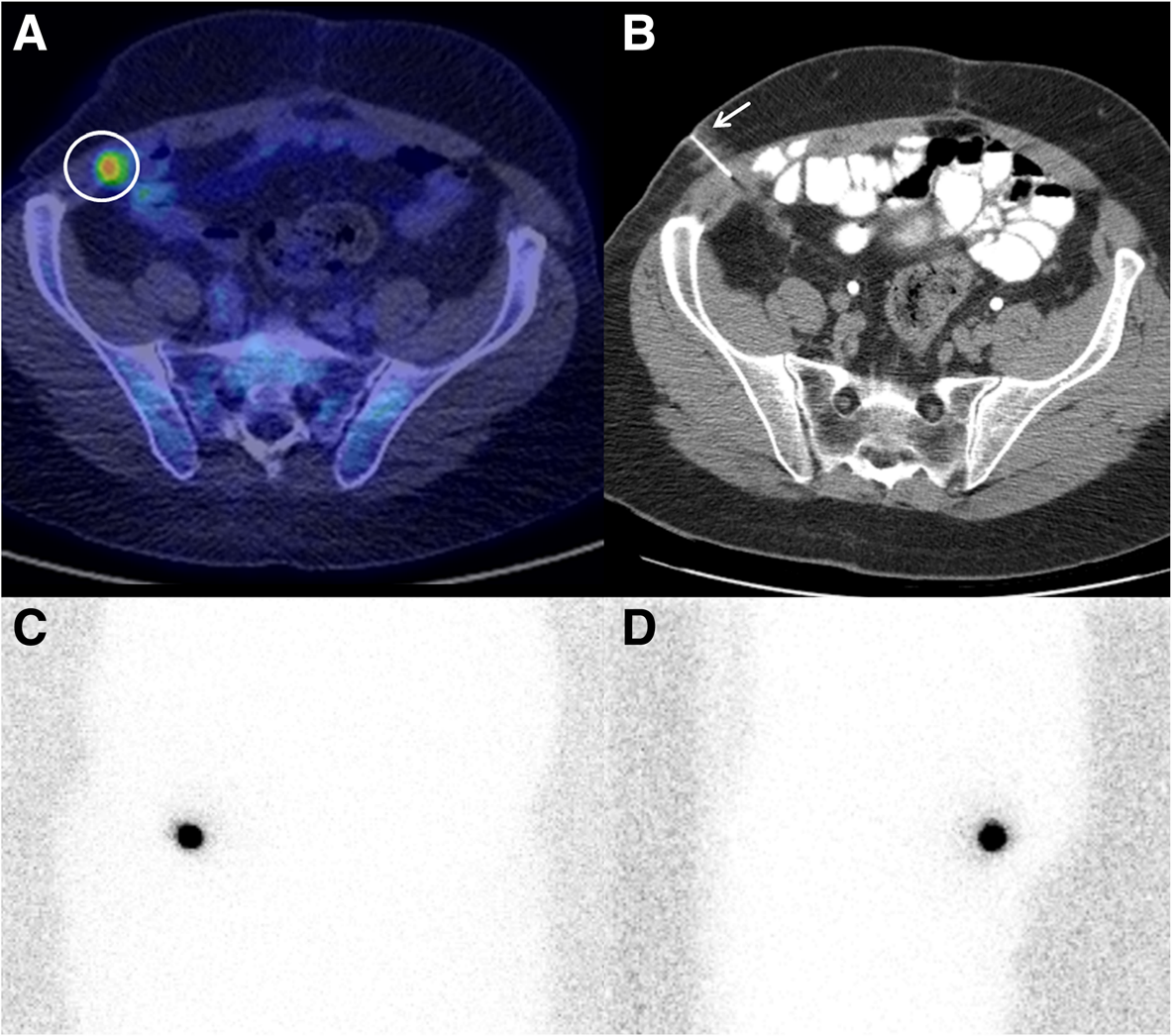
A 66-year-old male with right colon adenocarcinoma metastatic to the liver, status post right hemicolectomy, hepatic wedge resection, and chemotherapy. A follow-up FDG PET/CT (**a**) demonstrated a hypermetabolic nodule in the right lower quadrant abdominal wall (white circle) which was non-palpable. CT-guided (**b**) Tc-99m MAA was injected (white arrow) in the region of this hypermetabolic nodule. Anterior (**c**) and right lateral (**d**) scintigraphic views of the abdomen demonstrate excellent localization. This lesion was surgically resected and positive for metastatic adenocarcinoma

To our knowledge, we report the largest detailed analysis of atypical (non-breast and non-pulmonary) perilesional radiolocalizations. There are few case reports including endoscopic colonic and musculoskeletal lesions with similar favorable results [9–11]. The most common lesion site we found was rib (32%) which was likely due to expected intraoperative difficulty distinguishing the pathologic rib from adjacent normal ribs. Common themes arose in reviewing the clinical context of our cases. Several cases were localizations at sites of prior surgery (*n* = 7) and of lesions that were only evident by Nuclear Medicine molecular imaging (*n* = 8). Some cases had non-diagnostic or discrepant biopsy results (*n* = 4), failed to identify the lesion on prior surgery (*n* = 1), and had lesions underlying a large amount of soft tissue in obese patients (*n* = 2). We raise awareness to a technique that can successfully localize lesions which may otherwise be challenging to localize intraoperatively, such as non-palpable rib (Fig. 1), central mesenteric lymph nodes (Fig. 2), and abdominal wall lesions (Fig. 3).

The limitations of our study include a small number of subjects. While correlations between the data reviewed may exist, the statistical power of this study is inadequate. Qualitatively, the reported variations in injection and imaging parameters seemed to have little impact on the efficacy of radiolocalization. The cases with draining lymph node radiotracer uptake (lymph nodes adjacent to the site of injection) did not seem to interfere with surgical localization. Further, we regard the injection technique to be safe when performed by someone skilled in radiological interventional procedures.

Radiolocalization of atypical lesions may reduce surgical time and degree of surgical invasion for some patients. However, comparison of radiolocalized surgical excision with a control group is needed which poses a challenge since each case is often unique.

## Conclusions

Direct perilesional Tc-99m MAA injection can be used to aid surgeons in removing small lesions that are difficult to identify intraoperatively. The described injection and imaging parameters seem adequate to successfully localize a variety of atypical lesions for intraoperative removal using a hand-held gamma probe and may allow for more direct, minimally invasive surgical approaches.

## Data Availability

The datasets used and/or analyzed during the current study are available from the corresponding author on reasonable request.

## Abbreviations

CT: Computed tomography
Tc-99 m MAA: Technetium 99 m macroaggregated albumin
Tc-99 m SC: Technetium 99 m sulfur colloid
